# Survival and health economic outcomes in heart failure diagnosed at hospital admission versus community settings: a propensity-matched analysis

**DOI:** 10.1136/bmjhci-2022-100718

**Published:** 2023-03-15

**Authors:** Patrik Bachtiger, Mihir A Kelshiker, Camille F Petri, Manisha Gandhi, Moulesh Shah, Tahereh Kamalati, Samir Ali Khan, Gareth Hooper, Jon Stephens, Abdullah Alrumayh, Carys Barton, Daniel B Kramer, Carla M Plymen, Nicholas S Peters

**Affiliations:** 1 National Heart and Lung Institue, Imperial College London, London, UK; 2 Imperial College Healthcare NHS Trust, London, UK; 3 Imperial College Health Partners, London, UK; 4 Lighthouse Innovations Limited, London, UK; 5 Upstart Breakthrough Strategy Limited, London, UK; 6 Harvard Medical School, Boston, Massachusetts, USA

**Keywords:** Public Health, Primary Health Care, Patient Outcome Assessment, Outcome and Process Assessment, Health Care, Medical Record Linkage

## Abstract

**Background and aims:**

Most patients with heart failure (HF) are diagnosed following a hospital admission. The clinical and health economic impacts of index HF diagnosis made on admission to hospital versus community settings are not known.

**Methods:**

We used the North West London Discover database to examine 34 208 patients receiving an index diagnosis of HF between January 2015 and December 2020. A propensity score-matched (PSM) cohort was identified to adjust for differences in socioeconomic status, cardiovascular risk and pre-diagnosis health resource utilisation cost. Outcomes were stratified by two pathways to index HF diagnosis: a ‘hospital pathway’ was defined by diagnosis following hospital admission; and a ‘community pathway’ by diagnosis via a general practitioner or outpatient services. The primary clinical and health economic endpoints were all-cause mortality and cost-consequence differential, respectively.

**Results:**

The diagnosis of HF was via hospital pathway in 68% (23 273) of patients. The PSM cohort included 17 174 patients (8582 per group) and was matched across all selected confounders (p>0.05). The ratio of deaths per person-months at 24 months comparing community versus hospital diagnosis was 0.780 (95% CI 0.722 to 0.841, p<0.0001). By 72 months, the ratio of deaths was 0.960 (0.905 to 1.020, p=0.18). Diagnosis via hospital pathway incurred an overall extra longitudinal cost of £2485 per patient.

**Conclusions:**

Index diagnosis of HF through hospital admission continues to dominate and is associated with a significantly greater short-term risk of mortality and substantially increased long-term costs than if first diagnosed in the community. This study highlights the potential for community diagnosis—early, before symptoms necessitate hospitalisation—to improve both clinical and health economic outcomes.

**What is already known on this topic**

- Prompt, community-based diagnosis of heart failure (HF) is a public health priority for the NHS Long Term Plan, recognising that as of 2015, over 80% of all new HF diagnoses were made following an emergency hospital admission. To date, the clinical and health economic implications of hospital-based diagnosis have not been quantified.

**What this study adds**

- We performed a retrospective observational cohort study of patients receiving an index diagnosis of HF. We used propensity score matching to identify the prognostic impact of hospital admission-based diagnosis on mortality and performed cost-consequence analysis.

**How this study might affect research, practice or policy**

- Our findings can inform the planning for HF services. HF was diagnosed via hospital admission in 68% of patients. In the propensity-matched cohort, the ratio of deaths per person-months at 24 months was lower in those diagnosed in the community versus hospital, becoming statistically non-significant by 72 months. Diagnosis via hospital admission incurred an overall extra longitudinal cost of £2485 per patient.

## Introduction

Heart failure (HF) affects 5% of the population aged 75 years or older, with 60 000 new cases annually in the UK.^1^ There are multiple evidence-based therapies that improve survival and quality of life,^2 3^ with early dose optimisation associated with better clinical and health economic outcomes.^4 5^

Detection of HF via primary care is a priority in the National Health Service (NHS) Long Term Plan,^6^ recognising that historically, 80% of all new (index) diagnoses of HF were made via hospital admission.^7^ Hospitalisation with chronic HF is associated with increased hazard of death, repeat and prolonged hospitalisation,^8^ and the unit cost of such a hospital admission can exceed £10 000.^9^ Despite several initiatives to improve community-based detection of HF,^10^ only 4% of eligible patients complete the diagnostic pathway recommended by the National Institute for Health and Care Excellence (NICE) to time and target^7^— with overall minimal change in survival over the last decade.^11^

There are no recent estimates of whether hospitalisation continues to be the dominant pathway to index diagnosis. Relatedly, although hospitalisation around the time of HF diagnosis, and time-to-diagnosis^12^ may adversely affect survival, the underlying assumption that diagnosis through community pathways confers clinical and health economic benefits has not been investigated. Testing this assumption poses substantial methodologic challenges. Importantly, given the continually changing healthcare landscape, contemporary estimates of both survival and health economic burdens based on place-of-diagnosis are essential for shaping health policy interventions. This is now possible through linkage of contemporary, granular, real-world primary and secondary care clinical and cost data.^13–15^ The objective of this study was therefore to measure the combined prognostic and health economic impacts of different routes to index HF diagnosis.

## Methods

### Study design and data sources

We used a cohort study design following Strengthening the Reporting of Observational Studies in Epidemiology^16^ and 17REporting of studies Conducted using Observational Routinely-collected health Data (RECORD) checklists (online supplemental table 1).

10.1136/bmjhci-2022-100718.supp1 Supplementary data

We interrogated the Discover dataset within the Discover-NOW Trusted Research Environment, which pools depersonalised, contemporary, linked primary and secondary care electronic patient records from over 2.5 million patients in North West London (NWL). In addition to comprehensive demographic and clinical data, the dataset also captures health service utilisation and associated cost.^18^ The Discover dataset is accessible via Discover-NOW Health Data Research Hub for Real World Evidence through their data scientist specialists and information governance committee-approved analysts, hosted by Imperial College Health Partners.

### Cost data within discover

Primary care costs described a combination of General Medical Services, Personal Medical Services and Alternative Personal Medical Services contracts commissioned by NHS England and locally commissioned Clinical Commissioning Group schemes such as Local Improvement Schemes, Local Enhanced Services and Out of Hours Services. These costs reflected the actual outturn costs for historic years.

General practice (GP) level costs were apportioned across age groups based on historic analysis of appointment utilisation and then to patients, based on the number of recorded daily contacts that patients had with the practice. The cost allocation assumes that all patient contacts for the specified age group consume the same resource so all contacts will have the same unit price. This reflects the way contracts are commissioned by NHS England and locally commissioned schemes for GPs and hence recorded for charging purposes.

Hospital costs are based on actual activity and costs (ie, primarily cost per case) as reported by NHS Trust-issued patient-level service-level agreement monitoring reports. Some contractual adjustments, for example, emergency threshold adjustments, re-admission and other contractual penalties were applied retrospectively at the patient level. Costs do not reflect the sometimes-significant values that are not reported at patient level for example, direct access or contractual caps.

### Inclusion and exclusion criteria

**Figure 1:**
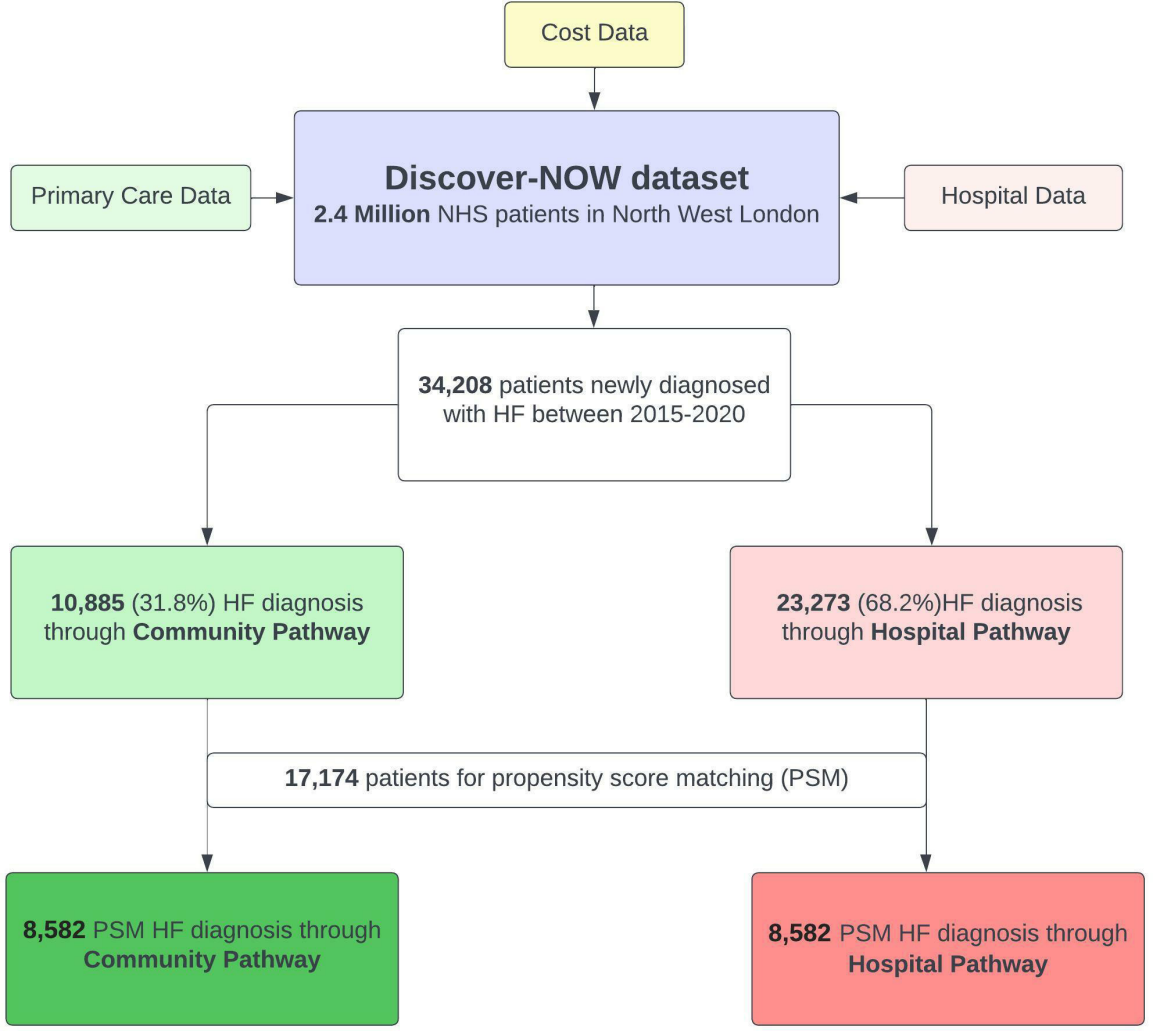
Flow diagram for selection of patients with HF for inclusion in the PSM analysis.

We included patients aged over 18 years old, with HF diagnosed between 1 January 2015 and 31 December 2020 (figure 1). We excluded patients aged 18 or under; those diagnosed with HF before 1 January 2015; and those who left the NWL area during the study period.

We considered specific ICD-10 codes for HF, in accordance with previous literature^7 11 19^ and by expert clinical consensus (online supplemental table 2). The clinical activity of the cohort was examined by identifying the first coded diagnosis of HF in the study period (index date) for each unique patient, and then mapping the healthcare resource utilisation of each unique patient during the study period. Two all-encompassing strata for routes to diagnosis were considered:

1. The ‘community pathway’ reflected HF diagnoses first coded within primary care records. This included patients diagnosed through specialist outpatient settings via primary care referral. We considered this the preferred route to HF diagnosis, in line with NICE guidance.^20^
2. The ‘hospital pathway’ reflected HF diagnoses made via an inpatient hospital admission. Such admissions were either non-elective (acute) or elective (eg, planned procedure). For both, we included those patients where, on discharge, HF diagnostic codes were listed as either a primary or secondary diagnosis. This was informed by clinical consensus since inconsistencies in clinical coding result in predominantly non-hierarchical coding of primary and secondary diagnoses.

### Patient characteristics

We identified comorbidities of interest, including chronic obstructive pulmonary disease (COPD), atrial fibrillation (AF), chronic kidney disease (CKD), ischaemic heart disease (IHD), stroke, type 2 diabetes mellitus (T2DM) and hypertension.

### Endpoints

The co-primary outcomes were all-cause mortality and cost consequence associated with index diagnosis of HF by hospital versus community pathway. More broadly, the overall differential split between hospital versus community pathway was also of interest.

### Data extraction, propensity score matching and statistical analysis

For the overall cohort and to facilitate comparisons between patients within each pre-specified strata, continuous variables are expressed as mean±SD and categorical variables as percentages. We performed χ^2^ tests, where appropriate, to examine differences in baseline clinical characteristics between patient pathways.

Time from diagnosis-to-death was captured for patients who died after the index date. Patient survival curves for mortality were constructed according to the Kaplan-Meier method for each pathway and compared by log-rank test.

We a priori assumed that there would be important clinical differences between our main analytic groups, driven by factors including different premorbid states and sociodemographic profiles. To characterise the independent association of route to diagnosis on outcomes, a propensity score was calculated using a logistic regression model to adjust for baseline differences in patient characteristics, and recognised predisposing factors for HF, including age, male gender, index of multiple deprivation rank, hypertension, AF, IHD, CKD and T2DM.

Importantly, to correct for differences in cost attributable to non-cardiovascular conditions, healthcare utilisation cost prior to HF diagnosis was also included in the model, for example, to account for imbalance of high-cost conditions such as cancer, costs for which are difficult to capture through matching by clinical codes. We performed a 1:1 comparison between nearest matching neighbours, using a caliper width of 0.2.

For cost-consequence analysis (a health economic evaluation methodology endorsed by the UK Government), healthcare utilisation cost was extracted from the index HF diagnosis date to the end of the study period, and included (not exclusively HF-related) primary care contacts, outpatient appointments, elective and non-elective admissions, and, where relevant, non-elective readmissions at 30 days. Patient level costs refer to the indicative spend calculated separately for each patient for each healthcare sector. A p value of <0.05 was considered statistically significant. Analyses were performed using open sourceRStudio (V.1.4.1717).

## Results

### Patient characteristics

Between 1 January 2015 and 31 December 2020, 34 208 patients received a diagnosis of HF (figure 1). This was split by 23 323 (68.2%) having this first recorded during a hospital admission (hospital pathway) and 10 885 (31.8%) in primary care (community pathway). Patient characteristics are summarised in table 1. The cohort diagnosed through hospital admission was older, had a higher representation of male sex and were more deprived. Four thousand six hundred and eighty-six (20.1%) were recorded in hospital as having HF as their primary diagnosis. The remainder recorded HF as a secondary diagnosis (online supplemental table 3). Thirty-one thousand and sixty-two (91.3%) of patients had at least one comorbidity at the time of HF diagnosis (online supplemental table 4). Two thousand seven hundred and thirty-eight (35%) of patients in the community pathway had at least one core HF symptom (breathlessness, ankle swelling, fatigue) recorded in primary care prior to the index date, compared with 4975 (65%) of patients in the hospital pathway (online supplemental table 5).

**Table 1:** Demographics and comorbidities for patients across total patient population diagnosed with heart failure in NWL in 2015–2020 (n=34 208) and PSM cohort (n=8582 for each of hospital and community pathway)

	Total population (n=34 208)		P value	Propensity-matched cohort (n=17 212)		P value
	Community pathway	Hospital pathway		Community pathway (n=8582)	Hospital pathway (n=8582)	
Age (SD)	72.26±13.50	73.84± (13.33)	<0.0001	73.53±12.18	73.61±12.62	0.66
Male gender (%)	5007 (46.0)	11 895 (51.1)	<0.0001	4644 (54.1)	4626 (53.9)	0.79
IMD (SD)	5.02±2.30	4.83±2.31	<0.0001	6.63±2.28	6.68±2.31	0.13
Ethnicity	–	–	<0.0001	–	–	0.92
Asian or Asian British (%)	2994 (27.5)	5913 (25.4)	–	2591 (30.2)	2567 (29.9)	–
Black or Black British (%)	948 (8.7)	1961 (8.4)	–	787 (9.2)	807 (9.4)	–
Mixed (%)	181 (1.7)	433 (1.9)	–	148 (1.7)	136 (1.6)	–
Other ethnic groups (%)	456 (4.2)	912 (3.9)	–	363 (4.2)	367 (4.3)	–
White (%)	5539 (50.9)	12 000 (51.5)	–	4693 (54.7)	4705 (54.8)	–
Unknown ethnicity (%)	767 (7.1)	2104 (9.0)	–	–	–	–
COPD (%)	1633 (15.0)	4234 (18.2)	< 0.0001	1470 (17.1)	1440 (16.8)	0.56
AF (%)	3592 (33.0)	7763 (33.3)	0.601851	2498 (29.1)	2571 (30.0)	0.23
CKD (%)	2939 (27.0)	6822 (29.3)	<0.0001	2338 (27.2)	2323 (27.1)	0.81
IHD (%)	4027 (37.0)	8704 (37.3)	0.564231	3862 (45.0)	3935 (45.9)	0.27
Stroke (%)	1013 (9.3)	2733 (11.7)	<0.0001	899 (10.5)	889 (10.4)	0.82
Ventricular arrhythmia (%)	130 (1.2)	281 (1.2)	0.933738	104 (1.2)	105 (1.2)	1.00
T2DM (%)	3592 (33.0)	8233 (35.3)	<0.0001	3099 (36.1)	3040 (35.4)	0.36
Hypertension (%)	7293 (67.0)	15 055 (64.6)	<0.0001	7001 (81.6)	6994 (81.5)	0.91

AF, atrial fibrillation; CKD, chronic kidney disease; COPD, chronic obstructive pulmonary disease; IHD, ischaemic heart disease; IMD, index of multiple deprivation; NWL, north west London; PSM, propensity score-matched; T2DM, type 2 diabetes mellitus.

### Propensity score-matched (PSM) cohorts

Prior to matching, the cohorts were unbalanced across most variables (table 1). Following PSM, the hospital and community pathway cohorts were matched across all variables of interest, including demographics, comorbidities, deprivation and healthcare utilisation costs before diagnosis of HF. The cohort consisted of 17 174 patients (8582 in each group).

### All-cause mortality

**Figure 2:**
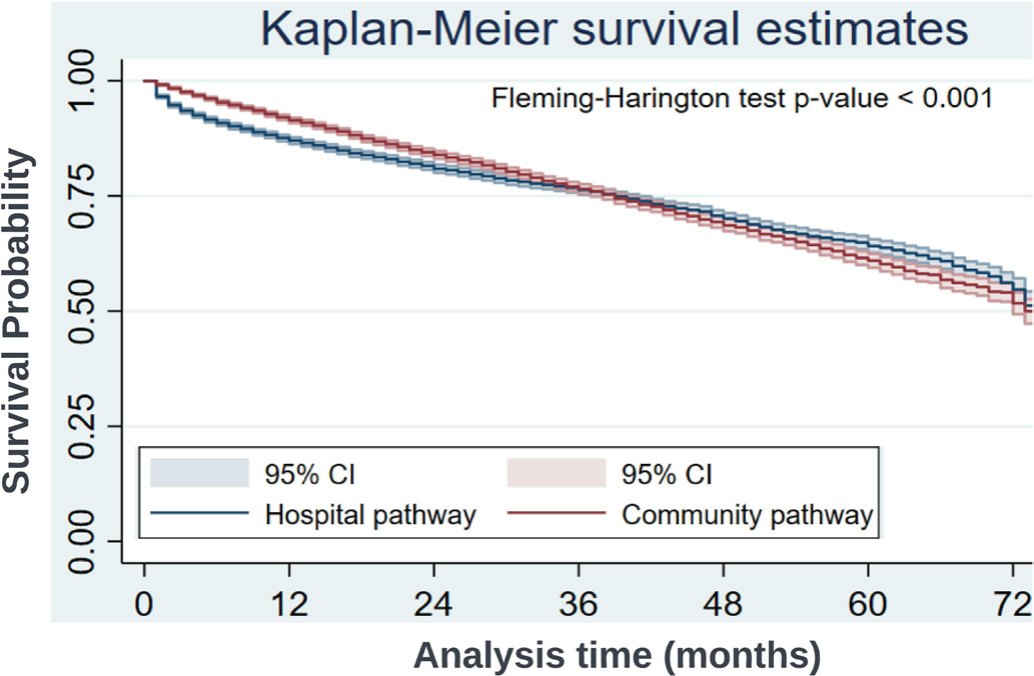
Kaplan-Meier survival estimates for all-cause mortality.

The median follow-up period was 29 months overall, 29 months for the hospital pathway and 30 months for the community pathway. At 24 months, the event rate for all-cause mortality in the hospital pathway cohort was 0.0094 per person-month, versus 0.0073 in the community pathway cohort (figure 2). Comparing community versus hospital diagnosis, the ratio of deaths per person-month at 24 months was 0.780 (95% CI 0.722 to 0.841, p<0.0001) (table 2). At 72 months, the event rate for all-cause mortality in the hospital pathway cohort was 0.0082 per person-month, versus 0.0079 in the community pathway (inter-pathway event ratio 0.960 (95% CI 0.905 to 1.020, p=0.18) (table 2).

**Table 2:** Event rate comparison between hospital and community pathways

Months	Community pathway		Hospital pathway		Rate ratio community:hospital (95% CI)	P value
	Patients at risk*	Death/person months	Patients at risk*	Death/person months		
24	5224	0.0073 (1215/166 255)	5040	0.0094 (1507/160 881)	0.780 (0.722 to 0.841)	<0.0001
48	2316	0.0076 (1920/252 467)	2145	0.0081 (1978/243 533)	0.936 (0.879 to 0.998)	0.04
72	254	0.0079 (2220/280 584)	223	0.0082 (2214/268 890)	0.960 (0.905 to 1.020)	0.18

*Number of patients alive/at risk of outcome of death

### Cost-consequence analysis

Table 3 shows cost-consequence analysis between the two cohorts, describing costs after the date of index HF diagnosis. Overall, in the PSM cohort, across all available metrics of health service utilisation, there was a £2485 longitudinal difference in cost associated with an HF diagnosis made through a community pathway versus hospital admission (table 3).

**Table 3:** Cost-consequence analysis post-index diagnosis of HF (propensity-matched cohort)

Category	Costs £ (mean±SD)						
	Non-elective	Elective	ED Cost	Outpatient	Primary care	Total cost	Difference
**Community £**	8714±16 409	3755±11 344	419±686	4901±7772	9508±20 240	27 298±29 470	–
**Hospital £**	10 804±17 719	4407±14 628	472±753	5341±8565	8759±20 954	29 783±32 264	+2485

ED, emergency department; HF, heart failure.

## Discussion

This study of a large population of NHS patients with HF demonstrates that, across a 6-year period, index diagnosis was predominantly through hospital admission. In a PSM cohort, index diagnosis of HF via hospital versus community pathway was associated with an increased rate of death in the first 24 months, with no difference between groups by 72 months. We found a substantial longitudinal cost saving (~£2500) following index HF diagnosis taking place through community pathways.

### Routes to diagnosis

Across the overall population of nearly 35 000 patients diagnosed with HF in NWL from 2015 to 2020, 70% were first diagnosed via hospital pathways. This proportion has changed little from the findings reported by Bottle *et al* (as cited in the NHS Long Term Plan), that between 2010 and 2013, 80% of HF diagnoses were first documented in hospital records.^7^ Our findings using data from 2015 to 2020 indicate that intervening efforts to improve community-based detection of HF have, at best, had modest impact. A substantial portion of HF is precipitated by acute disease, for example, myocardial infarction, requiring urgent hospitalisation. Such cases should not be counted as missed opportunities. However, previous studies found that among those diagnosed in hospital, the vast majority had also seen a GP in the previous year, with 37% having documented symptoms of HF.^14^ Notably, in our overall HF population there was no difference in the number of primary care encounters between the hospital and community pathway cohorts, with similar documented rates of HF symptoms prior to diagnosis (online supplemental table 5). This may represent heterogeneity in awareness of HF within primary care services, but may also highlight the non-specific nature of HF symptoms, which overlap with other common cardio-respiratory pathologies (eg, COPD)—a diagnostic challenge discussed in recent international guidelines.^2^ This is reflected in our unmatched study population, where multimorbidity (the presence of two or more long-term conditions) was more common among patients in the hospital pathway (online supplemental table 4).

### Prognostic association of route to diagnosis

We observed an early survival advantage associated with community pathway-based diagnosis to 24 months, which was not sustained at 72 months compared with hospital pathway-based diagnosis, reflecting the poor long-term prognosis in this condition. The mortality associated with HF is estimated to be between 53% and 67% five years after diagnosis with hospitalisation a known adverse prognostic marker in established HF.^11^ Consequently, the convergence of survival curves by 72 months may represent a ‘regression to the mean’ effect associated with heterogeneous adherence to gold standard therapy in both cohorts over a sustained period. Real-world estimates of adherence to gold standard therapy are low, with the most optimistic ranging from 40% to 60%, and declining as HF progresses.^21 22^ Translating the long-term protective effects observed in clinical trials requires dose optimisation, monitoring and patient concordance, and this could be better achieved if resource freed up by effective community diagnosis was channelled towards supporting patient adherence, compounded by the possibility that patients diagnosed via the community pathway were earlier in their HF disease course, and therefore more likely to realise the benefits of early initiation of prognostically beneficial therapies.^5^

Our findings are consistent with Taylor *et al*,^11^ who examined data from 2000 to 2017 to identify a patient’s first coded instance of an HF diagnosis in primary care records, and reported that those without hospital admission 3 months before or after diagnosis had better survival. A study reporting on data from 1997 to 2010 found significantly worse outcomes among patients where HF was only ever coded in hospital records and never registered in the primary care record.^19^

### Cost of heart failure

Across all health systems, the costs associated with HF are rising. However, detailed contemporary estimates on a per-patient level are lacking. To our knowledge, this is the first study to quantify the health economic opportunity of diagnosis through community pathways using a PSM cohort. Hospitalisation is the main driver of cost for HF.^23^ As might be expected, we found non-elective admission costs accounted for the majority (84%) of the long-term increased costs of patients in the hospital pathway. However, understanding the health economic burden of HF through units of hospitalisation has substantial limitations. Studies have shown that post-HF diagnosis, there is an average of one hospital admission per year, of which two thirds are attributable to non-cardiovascular comorbidities.^24^ However, we have shown that index HF diagnosis through hospital admission is unlikely to be coded hierarchically, that is, the primary diagnosis may be listed as common mimics and exacerbating conditions (eg, COPD, pneumonia) with new HF listed among the secondary diagnoses. Teasing apart the contribution of HF to the cost of each hospital admission is therefore challenging. More pragmatically, our study highlights that a community pathway-based diagnosis of HF offers an overall longitudinal cost-saving of £2500 per patient. This offers a compelling variable for cost modelling and an intelligible, robust metric for policymakers. Realising even a fractional increase in community diagnosis could release substantial cost savings and return on investment. In a simple example cohort of 10 000 patients (close to the number of patients diagnosed every 18 months in NWL), where 70% would otherwise be expected to be diagnosed through hospital admission, a reduction to 60% through increased community pathway activity could release a £2.5M saving.

### Strengths

The population of NWL represents a wide spectrum of sociodemographic inequality and includes the areas of highest ethnic diversity in the UK.^25^ To our knowledge, this is the first study to quantify the per-patient cost implications of route to index diagnosis of HF, adding a compelling health economic argument to the more established clinical rationale for investing in community diagnostic services.^7^ Notably our study accounted for a period of 6 years ending in 2020, and thus offers a contemporary picture of HF care, across a large population. This has been enabled using real-world primary care, secondary care, and cost data within the Discover dataset, as well as now routine systematic sharing of hospital discharge summaries to improve fidelity between primary and secondary care coding.

Not only were we able to extensively match on demographic and comorbidity profile, but also on cost before HF diagnosis. This will have controlled for potential confounding introduced by patients with extreme rates of service utilisation associated with rare and/or high-cost conditions; thereby specifically mitigating over-estimation of the benefits of community-based diagnosis due to the hospital cohort having higher costs before HF diagnosis.

### Limitations

The results of our study are best interpreted in the context of its limitations. Despite extensive propensity-score-matching across demographic, clinical and cost variables, some residual confounding is likely to remain and tempers our conclusions. The examination of real-world data is universally limited by the inconsistency and variable fidelity of medical coding in capturing variables of interest. HF is rarely coded with a granularity that describes preserved, moderately reduced or reduced ejection fraction. This may be addressed by future improvements in the coding of echocardiography results. Though this study interrogated granular clinical and cost data, substrata important to understanding a patient’s HF management were not available, for example, specific doses of disease modifying drugs that would have allowed inference beyond whether a patient was prescribed an HF-related medication, to whether this was optimised. Similarly, HF-specific quality of life metrics were not available for this cohort. Collectively this means we have likely been unable to fully account for differences in HF severity, where a degree of over-representation of severe disease in the hospital pathway may have skewed our observations. Lastly, though diverse in most other ways, the population of NWL does not encompass any rural/remote communities, whose experience of community versus hospital care may not be represented by our study.

### Opportunities

A recent study by Kahn *et al* searched primary care EHRs to identify a missed cohort of patients with HF, inviting them to a primary care-based HF service that enabled optimisation of prognostic medication and an increase in device prescription.^26^ Future research could quantify the clinical and health economic impacts of invited and/or opportunistic screening of an at-risk population identified through analysis of population-wide linked datasets. This approach, combined with emerging point-of-care testing technologies now reaching maturity^27^ could underpin a programme comparable to the NHS Diabetes Prevention Programme and the NHS Health Check. For example, artificial intelligence applied to a single-lead ECG may increase opportunistic detection by becoming integrated with commonly used tools such as stethoscopes.^27^ Given our finding that most patients had primary care encounters before their index HF diagnosis, many would have had a stethoscope examination, prompted by symptoms such as breathlessness. Ultimately, despite progress in therapies and evidence for best practice, the outlook for HF remains bleak, and community pathways may be best positioned to address this if powered by disruptive innovations that leverage integrated data and technology.

## Conclusion

Index diagnosis of HF through inpatient hospital admission continues to predominate and may be associated with a significantly increased short-term risk of mortality and substantially higher long-term cost compared with community pathways. This study highlights the need and opportunity for new approaches to increase community-based diagnoses, which would unlock longer, healthier lives for patients while substantially reducing HF cost burden.

## Data Availability

Data are available upon reasonable request.
