# Supplementary material for: Survival and health economic outcomes in heart failure diagnosed at hospital admission versus community settings: a propensity-matched analysis
Source: BMJ Health Care Inform. 2023 Mar 15;30(1):e100718. doi: 10.1136/bmjhci-2022-100718 (PMC10030479; doi:10.1136/bmjhci-2022-100718)
Supplement: Supplementary data [file bmjhci-2022-100718supp001.pdf]

Appendix

Online Supplementary Table 2

Read Code v2	ReadCodePreferredTerm30	Category
G58..	Heart failure	HF
662g.	New York Heart Association classification - class II	HF
662h.	New York Heart Association classification - class III	HF
G5yy9	Left ventricular systolic dysfunction	HF
G580.	Congestive heart failure	HF
G580.	Congestive heart failure	HF
585f.	Echocardiogram shows left ventricular systolic dysfunction	HF
662f.	New York Heart Association classification - class I	HF
G581.	Left ventricular failure	HF
G58z.	Heart failure NOS	HF
G5802	Decompensated cardiac failure	HF
G5yyC	Diastolic dysfunction	HF
G580.	Congestive heart failure	HF
G5yyA	Left ventricular diastolic dysfunction	HF
585g.	Echocardiogram shows left ventricular diastolic dysfunction	HF
G5801	Chronic congestive heart failure	HF
G5800	Acute congestive heart failure	HF
G583.	Heart failure with normal ejection fraction	HF
G583.	Heart failure with normal ejection fraction	HF
G581.	Left ventricular failure	HF
G582.	Acute heart failure	HF
G580.	Congestive heart failure	HF
G58..	Heart failure	HF
G584.	Right ventricular failure	HF
G581.	Left ventricular failure	HF
G5810	Acute left ventricular failure	HF
G5804	Congestive heart failure due to valvular disease	HF
21264	Heart failure resolved	HF
G581.	Left ventricular failure	HF
G58z.	Heart failure NOS	HF

G5yyB	Right ventricular diastolic dysfunction	HF
G5803	Compensated cardiac failure	HF
G58z.	Heart failure NOS	HF
G583.	Heart failure with normal ejection fraction	HF
G580.	Congestive heart failure	HF
SP111	Cardiac insufficiency as a complication of care	HF
G581.	Left ventricular failure	HF
1O1..	Heart failure confirmed	HF
G1yz1	Rheumatic left ventricular failure	HF
G232.	Hypertensive heart & renal dis with (congestive) heart failure	HF
G234.	Hypertension heart & renal dis+both (congestv) heart and renal fail	HF
G580.	Congestive cardiac failure	HF
G580.	Right heart failure	HF
G580.	Right ventricular failure	HF
G580.	Biventricular failure	HF
G58..	Cardiac failure	HF
G581.	Asthma - cardiac	HF
G581.	Impaired left ventricular function	HF
G58z.	Cardiac failure NOS	HF
14A6.	H/O: heart failure	HF
14AM.	H/O: Heart failure in last year	HF
SP111	Heart failure as a complication of care	HF
662i.	New York Heart Association classification - class IV	HF
G58z.	Weak heart	HF
G5y4z	Post cardiac operation heart failure NOS	HF
1J60.	suspected heart failure	HF
388D.	new york heart assoc classification heart failure symptoms	HF
G210.	malignant hypertensive heart disease	HF
G2101	malignant hypertensive heart disease with ccf	HF
G2111	benign hypertensive heart disease with ccf	HF
G21z1	hypertensive heart disease nos with ccf	HF
G230.	malignant hypertensive heart and renal disease	HF
G41z.	chronic cor pulmonale	HF
G5540	congestive cardiomyopathy	HF

G5540	congestive obstructive cardiomyopathy	HF
G5571	beriberi heart disease	HF
R2y10	[d]cardiorespiratory failure	HF
**ICD10 diagnosis**		
I500 - Congestive heart failure		
I501 - Left ventricular failure		
I509 - Heart failure, unspecified		
I420 - Dilated cardiomyopathy		
I255 - Ischaemic cardiomyopathy		
I110 - Hypertensive heart disease with (congestive) heart failure		
I429 - Cardiomyopathy, unspecified		

Online Supplementary Table 2. Discover-NOW and ICD-10 codes for heart failure.

Online Supplementary Table 3

ICD10 diagnosis	Number of patients	% of patients
J181 - Lobar pneumonia, unspecified	1261	7%
I214 - Acute subendocardial myocardial infarction	759	4%
I251 - Atherosclerotic heart disease	676	4%
J189 - Pneumonia, unspecified	639	3%
I489 - Atrial fibrillation and atrial flutter, unspecified	538	3%
A419 - Sepsis, unspecified	420	2%
N390 - Urinary tract infection, site not specified	335	2%
J440 - Chronic obstructive pulmonary disease with acute lower respiratory infection	310	2%
I249 - Acute ischaemic heart disease, unspecified	301	2%
R074 - Chest pain, unspecified	280	1%
J22X - Unspecified acute lower respiratory infection	277	1%
I210 - Acute transmural myocardial infarction of anterior wall	272	1%
N179 - Acute renal failure, unspecified	252	1%
J690 - Pneumonitis due to food and vomit	196	1%
R296 - Tendency to fall, not elsewhere classified	184	1%
I48X - Atrial fibrillation and flutter	177	1%
L031 - Cellulitis of other parts of limb	175	1%
U071 - Emergency use of U07.1	173	1%
I211 - Acute transmural myocardial in farction of inferior wall	172	1%
R060 - Dyspnoea	163	1%
Other	11,278	60%

Grand Total	18,838	100%

Online Supplementary Table 3. Primary diagnosis of patients diagnosed with heart failure coded as their secondary diagnosis.

Online Supplementary Table 4

	Total	Community Pathway	%	Hospital Pathway	%	p-value
**Total number of comorbidities**						<0.0001
**0**	-	936	9	2020	9	
**1**	-	2479	23	4822	21	
**2**	-	3073	28	6497	28	
3	-	4385	40	9808	42	

Online Supplementary Table 4. Prevalence of multiple comorbidities in each diagnostic pathway.

Online Supplementary Table 5

	Total	Community Pathway	%	Hospital Pathway	%	p-value
Patients with at least one HF symptom recorded in primary care prior to index date	7713	2738	35	4975	65	3.65
Patients with no symptoms of HF recorded in primary care prior to index date	26305	8135	31	18170	69	

Online Supplementary Table 5. Prevalence of HF symptoms prior to index date.

Online Supplementary Table 6

Health service utilisation	Community	Hospital
ED attendances (before diagnosis)	£297	£398
ED attendances (after diagnosis)	£457	£552
Diagnosis spell in hospital	-	£4,207
Non-elective admission (before diagnosis)	£3,328	£4,397
Non-elective admission (after diagnosis)	£6,699	£8,749
Day case elective procedure (before diagnosis)	£1,165	£1,276
Day case elective procedure (after diagnosis)	£1,335	£1,358
Elective admission before diagnosis	£3,189	£2,788
Elective admission before diagnosis	£3,365	£3,572
Outpatient (first visit) before diagnosis	£418	£457
Outpatient (first visit) after diagnosis	£496	£504
Outpatient (follow-up) before diagnosis	£335	£443
Outpatient (follow-up) before diagnosis	£396	£411
Outpatient procedure (before diagnosis)	£367	£406
Outpatient procedure (after diagnosis)	£390	£389
Primary care encounters (before diagnosis)	£4471	£4,876
Primary care encounters (after diagnosis)	£4869	£2,136
**Total**	£29,864	£36,919
**Difference**		**£7,055**

Online Supplementary Table 6. Non-propensity matched cost consequence analysis by diagnostic pathway.
